# Nutrient limitations alter cell division control and chromosome segregation through growth-related kinases and phosphatases

**DOI:** 10.1098/rstb.2011.0124

**Published:** 2011-12-27

**Authors:** Mitsuhiro Yanagida, Nobuyasu Ikai, Mizuki Shimanuki, Kenichi Sajiki

**Affiliations:** The G0 Cell Unit, Okinawa Institute of Science and Technology (OIST) Promotion Corporation, Tancha 1919-1, Onna, Okinawa 904-0412, Japan

**Keywords:** Tor1, Tor2, Ssp1, protein phosphatase, separase, starvation

## Abstract

In dividing fission yeast *Schizosaccharomyces pombe* cells, the balance between Wee1 kinase and Cdc25 phosphatase which control the cyclin-dependent kinase (CDK) at the G2–M transition determines the rod-shaped cell length. Under nitrogen source starvation or glucose limitation, however, cell size determination is considerably modulated, and cell size shortening occurs for wild-type cells. For several mutants of kinases or phosphatases, including CDK, target of rapamycin complex (TORC) 1 and 2, stress-responsive mitogen-activated protein kinase (MAPK) Sty1/Spc1, MAPK kinase Wis1, calcium- and calmodulin-dependent protein kinase kinase-like Ssp1, and type 2A and 2A-related phosphatases inhibitor Sds23, this cell shortening does not normally occur. In *tor1* and *ssp1* mutants, cell elongation is observed. Sds23 that binds to and inhibits 2A and 2A-related phosphatases is synergistic with Ssp1 in the cell size determination and survival under low glucose and nitrogen source. Tor2 (TORC1) is required for growth, whereas Tor1 (TORC2) is needed for determining division size according to different nutrient conditions. Surprisingly, in growth-diminished *tor2* mutant or rapamycin-treated cells, the requirement of separase/Cut1-securin/Cut2 essential for chromosome segregation is greatly alleviated. By contrast, defects of *tor1* with secruin/*cut2* or overproduction of Cut1 are additive. While Tor1 and Tor2 are opposite in their apparent functions, both may actually coordinate cell division with growth in response to the changes in nutrients.

## 1. Introduction

In the book ‘The biology of the cell cycle’, Mitchison [1] wrote that one of the basic questions to ask about the cell cycle is what is the pattern of overall cell growth between one division and the next. This question still remains. It is surprising how little we know about the basic mechanism of cell growth in the relationship to the cell cycle. Mitchison pointed out the criteria that can be used for measuring cell growth; they are cellular volume, constituents including water, total dry mass, macromolecular dry mass and low molecular weight compounds pool, protein and other macromolecules such as RNA, DNA and carbohydrates. Precise measurements of these parameters have actually not advanced well technically since the time of writing the book, so that growth data are often missing or not satisfactory in many cell cycle studies. The fission yeast *Schizosaccharomyces pombe*, a eukaryotic micro-organism, is convenient for estimating the cell volume by simply measuring cell length as its cell shape in the vegetative phase is rod-like, so Mitchison pioneered the use of *S. pombe* as a eukaryotic model for understanding growth versus cell cycle. The growing phase (e.g. cell length increase) of *S. pombe* in the standard (rich) culture medium occurs after DNA replication, whereas the cell length is constant during the phases of mitosis and cell division [2,3].

Thuriaux *et al*. [4] and Nurse & Thuriaux [5] isolated *S. pombe* mutants that were thought to be altered in the control coordinating cell division with cell growth. More than 50 mutant strains—most severely altered in this control—were isolated, which showed the same growth rate as wild-type, but divided at a much shorter cell size. The great majority of the mutants were genetically mapped within the single *wee1* locus (wee means little), and the remaining one mutant turned out to be an allele of *cdc2*, originally called *wee2*. At that time, Wee1 and Cdc2 were predicted to be involved in a control initiating mitosis when the cell attains a critical cell length. The *wee1*^+^ gene was postulated to code for a negative element or inhibitor, and *cdc2*^+^ to code for a positive element or activator in the mitotic control. We now know that these elements are indeed cell cycle-regulating protein kinases; Wee1 directly phosphorylates Cdc2 and inhibits the kinase activity. The original mutation *wee2-1* (*cdc2-w1*) escapes the negative regulation by Wee1 resulting in premature division with regard to cell size. The success in identifying Wee1 as the negative regulator of mitotic entry came from the genetic screen for mutants displaying the strongest *wee* phenotype. In addition, the growth rate was shown to be normal in these mutants, separating the growth issue from the cell cycle control. In retrospect, there were a number of mutants that showed the semi-*wee* phenotypes, which were wisely not investigated at that time. After 30 years since the discovery of *wee1* mutants, however, the time may be ripe to shed light on broad mutations that produce the less severe, ‘wee-like’ phenotypes, many of which may include the defects in growth *and/or* cell cycle control.

Cdc25, another important regulator for mitotic entry, was discovered by Fantes [6] through the analysis of interactions between *wee* and various *cdc* (cell division cycle) mutants. The block of mitotic entry or the prolonged G2 interphase caused by a defective *cdc25* allele is suppressed when combined with the *wee* mutants. Suppression of the temperature-sensitive (ts) *cdc25* phenotype by *wee1* is almost complete. Other *cdc2-w* mutations (e.g. cdc2-3w) are sensitive to Wee1 function, but largely abolish Cdc25 requirement. Cdc25 turned out to be a protein phosphatase [7,8] that competes with Wee1 and is an activator of Cdc2 by dephosphorylating the tyrosine residue (Y15) of Cdc2. Not only *cdc25*, *cdc13* (mitotic cyclin mutant) and most *cdc2* ts alleles are blocked at the boundary of G2–M transition. Note that the loss of Cdc25 and Cdc2–Cdc13 blocks mitotic entry but not cell growth, leading to the formation of highly elongated cells arrested in the G2–M boundary but continuing growth. The loss of cyclin-dependent kinase (CDK) activation disrupts the cell cycle control and also affects the cell size determination as clearly exemplified by *wee1* mutation. It is obvious, though often forgotten, that the cell size is strongly affected by cell cycle control, growth control or both. In *wee1* mutant cells, growth is not inhibited, but prematurely committed mitosis and following cytokinesis take precedence over growth to produce small cells.

## 2. Extensive shortening of cell size occurs by division under nitrogen deficiency

**Figure 1.**
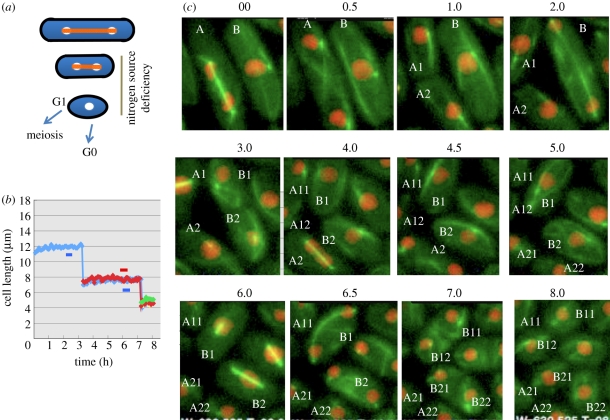
Nitrogen source deficiency-induced cell size shortening of wild-type. (*a*) *Schizosaccharomyces pombe* wild-type cells under the absence of nitrogen source (NH_4_Cl) divide twice and arrest at a temporal G1 phase followed by meiosis or the entry into quiescent G0 phase dependent on the presence or absence of mating. The orange bar represents the spindle. (*b*) Cell length is shortened dramatically during two divisions after transfer (time 0) from the complete EMM2 to the NH_4_Cl-deficient EMM2 medium. (*c*) Light micrographs of time course changes of the two cells in the complete medium (time 00 h) shifted to the nitrogen-deficient medium for 8.0 h. The first division produced daughter cells A1, A2 and B1, B2 cells. The second division produced A11, A12, A21, A22 and B11, B12, B21, B22 cells. Red, histone H2A; green, tubulin and the Sid4 SPB protein (see text).

Wild-type *S. pombe* cells respond to nutritional change by changing the cell size. When *S. pombe* is transferred from the complete synthetic Edinburgh Minimal Medium (designated EMM2) to EMM2 –N lacking the nitrogen source (NH_4_Cl), cells can divide approximately twofold, an approximately fourfold increase in number in the absence of the growth phase, producing short and round cells, which are arrested at the G1 phase (figure 1*a*,*b*; [9–12]). Note that the EMM2 medium has no amino acids, so that NH_4_Cl is the sole nitrogen source. These divisions in EMM2 –N are thought to occur and resulting quiescent cells are maintained through recycling the intracellular nitrogen sources. If cell populations are competent for meiosis in the EMM2 –N medium, then cells exit from the G1, conjugate with other mating type cells, and irreversibly commit meiotic divisions. If cell populations are heterothalic (mono-sexual), however, then solitary cells enter the G0 phase at around 12 h and remain viable in the quiescent phase for quite long times (greater than one month). The capability to mate with opposite mating type cells is lost upon entry into the G0 phase. The size of small G0 cells remains constant. The nitrogen-starvation-induced G0 cells thus lack both growth and division, but are metabolically active [12,13].

During nitrogen source deficiency (designated N-starvation hereafter)-induced divisions, the reduction of cell size occurs from the average 12 µm long rod of vegetative cells in EMM2 to 5 µm diameter round-shaped G0 phase cells in EMM2 –N (figure 1*b*). The short bars in figure 1*b* represent the timing of mitosis prior to the first and second cell division. Cell length shortening from mother (blue) to daughter (blue, red) and grand-daughter (red, green) is clearly shown. Note that the growth phase (cell length increase) is missing in these divisions. The calculated ellipsoidal cell volume and the protein content measured for these small G0 cells are 1/3 and 1/6, respectively, of those of vegetative cells [14]. These small G0 cell features are not called the *wee* phenotype as they are seen in N-starvation, and, besides, cells are arrested while the *wee1* mutant cells grow and divide. G0 cells are round-shaped, suggesting that the cytoskeletal architecture is altered from that of *wee1* in the presence of nitrogen source. Having stated these differences, there may exist some parallel: accelerated mitoses occur twice in the EMM2 –N medium prior to the arrest. Hence, during nutrient deficiency, wild-type cells try to make a final division before quiescence, which occurs prematurely at a time when the cell size is still small. The *wee* phenotype therefore may represent the commitment of mitosis under starvation conditions. In other word, N-starvation can produce *wee*-like cells.

Microarray and high-resolution analysis of transcripts indicate that one half of approximately 5000 whole genome genes had significantly changed levels by simply removing NH_4_Cl from the medium [14,15]. Mitosis under N-starvation was analysed by movies (figure 1*c*). Two example of living cells (A and B) were observed by colour-tagged histone H2A-RFP (red, chromatin), alpha-tubulin-GFP (green, microtubule; [16]) and Sid4-GFP protein (green) localized at the spindle pole body (SPB; [17]). They divided at a time interval of approximately 3 h and were arrested (e.g. B11, B12, B21 and B22). The mode of cell division was normal except for the absence of growth, and the decline of cell length by division. The anaphase spindle was short owing to the shortened cell length, or occasionally elongated as the curved form.

How is Wee1 kinase involved during the nitrogen-deficiency-induced ‘premature’ division? Paradoxically, Wee1 kinase is required for the meiotic entry under N-starvation, and phosphorylates the Y15 residue of Cdc2 (CDK1) kinase [18]. The activity of Wee1 is thus not lost. Hence, while not identified, other kinases and phosphatases may be involved in cell size shortening under N-starvation. The state of Cdc2 kinase in the G0 phase is presumably inactive with abundant Rum1, an inhibitor of Cdc2 [14,19], and by Cdc2 Y15 phosphorylation [20].

## 3. Sty1 and Ssp1 in addition to Cdc2 are required for shortening under N-starvation

To identify mutants that are defective in cell size shortening under N-deficiency, 600 ts strains were searched for any that remained rod-shaped while the cells divided [13]. Only six classes of mutants remained rod-shaped while they divided before the arrest at 26°C, the permissive temperature. Genetic analyses indicated that these have mutations in protein kinase genes that are implicated in cell cycle control and stress-responsive signalling [13]: mitogen-activated protein kinase (MAPK) *sty1/spc1-989*, MAPK kinase (MAPKK) *wis1-558,-982*, CDK *cdc2-974*, mitotic cyclin *cdc13-563*, calcium- and calmodulin-dependent protein kinase kinase (CaMKK)-like *ssp1-412*. In databases, p38-like MAPK Sty1 and MAPKK Wis1 are classified as involved in cell cycle, cell growth, gene expression, translation and cellular response to stress, whereas Ssp1 is involved in cell cycle, cell growth, cell morphology, actin cytoskeleton and response to stress. Under N-starvation, stress-responsive Wis1 and Sty1 and calcium-, calmodulin-dependent Ssp1 kinases are thus coordinated with the cell cycle regulator Cdc2–Cdc13 to appropriately change the cell size and shape.

**Figure 2.**
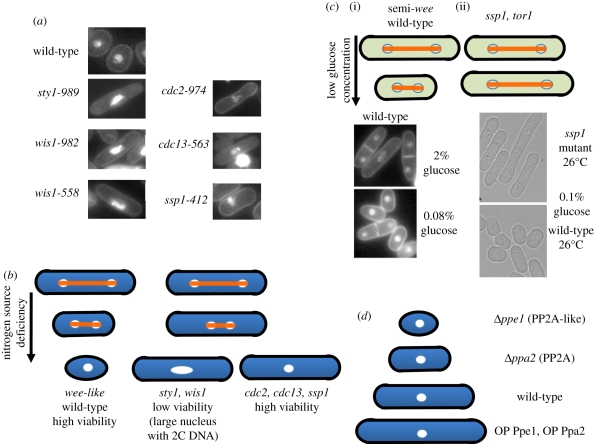
Genes required for cell size shortening under limited nitrogen or glucose. (*a*) In the absence of nitrogen source, mutant cells *sty1-989*, *wis1-982*, *-558*, *cdc2-974*, *cdc13-563* and *ssp1-412* remained rod-shaped at 26°C, the permissive temperature. (*b*) Schematic of mutants that fail to shorten cell size upon the transfer to the culture deficient of the nitrogen source. (*c*) Length of dividing wild-type cells is shortened in EMM2 medium containing low (0.1%) glucose instead of standard 2% glucose concentration. Mutant *ssp1-412* is elongated in 0.1% glucose at 26°C rather than the shortening in wild-type. A similar result is obtained for *tor1* mutant cells at the semi-permissive temperature. (*a*,*b*) Based on Sajiki *et al*. [13]; (*c*) based on Hanyu *et al*. [21]. (*d*) The phosphatase deletion mutant *Δppe1* or *Δppa2* results in the production of small, round or short semi-*wee* cells, respectively. By contrast, overproduction of Ppe1 or Ppa2 causes the semi-*cdc25* elongation phenotype [22,23].

Examples of cells in the nitrogen-deficient medium are shown in figure 2*a* in comparison with the wild-type control. Interestingly, mutants *sty1* and *wis1* retained high viability immediately after the two rounds of divisions, but greatly lost the viability during the transition from the transient G1 to the quiescent G0 phase [13]. Resulting long rod-shaped *sty1* and *wis1* mutant cells displayed large nuclei (figure 2*b*). Other mutants (*cdc2, cdc13 and ssp1*) also revealed rod-shaped cells, but retained high viability in the G0 phase at the permissive temperature, suggesting that being rod-shaped *per se* during the divisions under N-starvation does not cause the loss of viability.

Nevertheless, these different classes of protein kinases are vital for shortening divisions under N-starvation. It is thus plausible to speculate that Ssp1, MAPK (Sty1) and MAPKK (Wis1) may directly or indirectly regulate the activation of Cdc2 to cause accelerated mitosis under N-starvation. In the published movies of *sty1-989* and *wis1-982* mutant cells, the growth phase clearly exists during the division cycles in EMM2 –N medium, while cells keep their rod shape [13]. Sty1 and Wis1 are thus essential for the cessation of growth in N-starvation. It is truly surprising how *sty1*- and *wis1*-deficient cells manage to commit divisions while keeping an apparent growth phase without an outsource of nitrogen. One possible reason for such aberrant growth might be due to the use of an abundant carbon source (glucose) for cell size increase, while a nitrogen source may be available by recycling. Indeed, the rate of glucose consumption is high in *sty1* mutant cells (L. Uehara & A. Mori 2011, unpublished data). Based on the reason why MAPK and MAPKK mutants post-division lose their viability after entry into the G0 phase, we suspect that the remodelling of nuclear chromatin required for the survival during long-term G0 quiescence may not occur, because the nuclei in these mutants were abnormally expanded [13].

There have been a number of reports showing the close relationships between *S. pombe* CDK (Cdc2–Cdc13) and stress-responsive pathway (SRP) through various genes, such as protein kinase A (PKA), response regulator Mcs4, Polo-like kinase Plo1 and target of rapamycin (TOR) kinase [24–29]. Plo1 may be important in cell size shortening, as it regulates cytokinesis [30–32]. Petersen & Nurse [27] reported that TOR kinase, which is modulated by nutritional conditions and inhibited by rapamycin, controls the entry into mitosis through stress-responsive Sty1. However, overall understanding of the relationships among CDK, SRP and TOR is still meagre. It is crucially important to set up a clear-cut experimental system to relate growth with the cell cycle. In this regard, the growth-absent, size-shortening division under N-starvation is an excellent model to understand the suppression of growth by nutritional stress. This system may resemble the early embryonic egg cleavage.

## 4. Under low glucose, the semi-*wee* is induced in wild-type but not in two kinase mutants

Glucose is a source of energy for cells as well as the source of cell structure, and the cellular mode of its utilization may be centrally important for understanding cell growth, division and quiescence. We investigated the cell division–quiescence behaviour of *S. pombe* under diverse glucose concentrations from excess, regular diet and starvation to fasting (111–0 mM; [33]). The division mode (observed under a microscopic perfusion system that constantly supplied the medium) was surprisingly normal except for the shortening of cell length (20–30%) when glucose concentrations were highly diluted (5.6 mM, 1/20 concentration of the standard culture medium that contains 111 mM (2%) glucose). This semi-*wee* length-shortening property is observed in a range of low glucose levels equivalent to human blood sugar concentrations (figure 2*c*(i)). Normal human blood glucose content is around 4 mM (0.08%) before breakfast. *Schizosaccharomyces pombe* may be a good model, with regard to understanding the cellular uptake and utilization mechanism of normal blood glucose, which is defective in certain patients with type II diabetes.

When glucose concentration is further reduced to a level of starvation, the nature of division becomes stochastic in addition to cell shortening, accompanied by a curious epigenetic inheritance of division timing. A sharp transition from division to quiescence takes place in a narrow glucose concentration range (from 2.2 to 1.7 mM). Under severe glucose starvation (1.1 mM), cells are mostly quiescent and only a small population of cells divide. Under fasting condition (0 mM), division is immediately arrested, and fasting cells have a short chronological lifespan (16 h) if the shift was abrupt. If, however, the shift to fasting is slow, then the resulting lifespan greatly increases. Various biomarker compounds specific for different glucose concentrations have been identified. Glucose concentrations thus control the cell size, the doubling time, the uniformity of cell division pattern and even epigenetic behaviour among different cell lineages.

It is surprising to find that the doubling time in 111 and 4.4 mM glucose is the same, but the semi-*wee* phenotype, 20–30% reduction in cell size, partly explains the non-prolonged doubling time in low glucose. Taken together, *S. pomb*e has a very wide range of optimal glucose concentrations for the rate of division with regard to the doubling time. *Schizosaccharomyces pombe* under low glucose may thus sacrifice the growth phase in order to keep the same rate of increase in cell number. Here, the linked regulation between growth and division clearly exists. Note that glucose limitation or starvation, or even fasting does not affect cell shape, whereas nitrogen starvation causes the deviation of cell shape (to round) from rod.

A subsequent question is what kind of gene function converts the information about limited glucose to cell size determination. Hanyu *et al*. [21] reported that, under low glucose concentrations, Ssp1 kinase described above plays an important role in the size control; mutant *ssp1* cells remain long rod in approximately 0.1 per cent glucose (2% in the regular medium) at 26°C, the permissive temperature, displaying the semi-*cdc25* phenotype (figure 2*c*(ii)). At the semi-permissive temperature under low glucose, the phenotype becomes severer as mutant cells fail to divide. Furthermore, the measurements of remaining glucose concentrations in the medium showed that the rate of glucose consumption is considerably slower in *ssp1* mutant cells than that of wild-type under limited glucose, suggesting that the utilization of glucose is impaired in *ssp1* mutant cells. Note that *ssp1* cells fail to reduce cell size under both nitrogen and glucose limitation, so that it may have a broad role for the utilization of nutrients, such as in incorporation or transport of nutrients. Indeed, Ssp1 is the cell cortex protein. Ssp1 kinase may function in parallel with Gsk3 kinase and oppose PP2A and PP2A-related phosphatases [21].

Another protein kinase identified is Tor1, the catalytic subunit of TORC2 kinase, the mutant of which fails to reduce the cell length under limited glucose (figure 2*c*; [20]). A new *tor1* substitution mutant *tor1-L2045D* was constructed using the information of the ts *tor2* mutation site that resides in the highly conserved phosphatidyl inositol kinase domain of the catalytic subunit [34]. Only the substitution mutant *tor1-L2045D* displayed the ts phenotypes among the five different substitutions made at the same site. This *tor1-L2045D* mutant (*tor1-D* hereafter) grows normally at 26°C but fails to grow at 36°C or under low glucose concentration at the semi-restrictive temperature, displaying semi-*cdc25* phenotype at 36°C or in low glucose at a semi-permissive temperature (the deletion mutation *Δtor1* is more severe than the *tor1-D* mutation). Taken together, two nutrient-sensitive protein kinases Ssp1 and Tor1 are responsible for the cell size reduction in response to limited glucose. Screening a large number of ts and deletion mutants grown under the low glucose identified these mutants (details described elsewhere).

## 5. CaMKK-like Ssp1 is related to SIT4-like Ppe1 phosphatase, cortex actin and AMPK-like Ssp2 kinase

The *ssp1*^+^ gene was originally identified as one of the extragenic ts suppressors for the cold-sensitive (cs) deletion phenotypes of Ppe1 [35]. Ppe1 is a member of the evolutionarily conserved type 2A-related phosphatase family, similar to budding yeast SIT4 and mammalian PP6 [36–39]. Ssp1 kinase is involved in salt–stress responses as it is rapidly recruited to the plasma membrane during high salt-induced osmotic pressure [32,40]. While the mutant phenotype resembles that of *sty1* stress-responsive MAPK mutants, Ssp1 and Sty1 do not seem to act through the same pathway. Ssp1 controls the state of cortical actin [32,35,40]: *ssp1* mutant cells grow in a monopolar fashion and arrest at the G2–M boundary, but the relationship between Cdc2 and Ssp1 in the cell cycle is unclear. Cortical actin distribution in growing *ssp1* mutant cells is also monopolar. Ssp1 is hence required to promote the bipolar, rather than monopolar, cell elongation. Overproduction of Ssp1 kinase caused the dispersion of actin, resulting in round cell shape [35]. By contrast, the *Δppe1* deletion mutant causes the dispersal of actin, resulting in small round cells. Judging from the mutant phenotypes, Ssp1 kinase and Ppe1 phosphatase may be opposing. They might act on the same substrate important for responding to nutrient limitation. One candidate is Ssp2 (one of the two AMPKs, see below).

Ssp2 kinase was also identified as an extragenic suppressor for *Δppe1* [35]. Ssp2 is an *S. pombe* homologue of mammalian AMP-dependent protein kinase (AMPK) and budding yeast Snf1, containing three distinct subunits [21]. AMPK is thought to be a central player for carbohydrate catabolic processing. *Schizosaccharomyces pombe* actually has two AMPK-like catalytic subunits, Ppk9 and Ssp2, and 1 β and 1 γ subunit homologues (Amk2/Spcc1919.03c and Cbs2, respectively). The regulatory subunit Cbs2 is essential to maintain the viability of N-starved G0 cells. The cell cycle phenotypes of AMPK catalytic subunit mutants remain to be investigated.

## 6. Roles of PP2A Ppa2 and PP2A-related Ppe1 phosphatases for mitotic entry and cell size control

It has been known that PP2A and PP2A-related catalytic subunits, Ppa2 and Ppe1, respectively, affect the size of dividing *S. pombe* cells [22,23]. The cs phenotype of the *Δppe1* mutant is rescued by overproduction of the catalytic subunits of PP2A. Both *Δppe1* and *Δppa2* mutants are small, but differ in shape, round and rod, respectively (figure 2*d*). *Schizosaccharomyces pombe* has the second PP2A catalytic subunit Ppa1. The double mutant *Δppa1 Δppa2* is lethal, producing the small rod cells indistinguishable from *wee1* [41]. If okadaic acid is added to the culture of single mutant *Δppa2*, then basically the same result was obtained. By contrast, overproduction of Ppa2 results in the semi-*cdc25*-like elongation, producing long rod cells. Taken together, PP2A and PP2A-related phosphatases may play similar roles in the cell size control. Judging from cell shape of the mutants, *Δppe1* might be more related to growth defect, whereas *Δppa2* is defective in mitotic entry. Kinoshita *et al*. [22] showed that Ppa2 interacts genetically with the cell cycle regulators Cdc25 tyrosine phosphatase and Wee1 kinase in *S. pombe*: the *Δppa2* mutant is lethal when combined with *wee1-50*, but partially suppresses the phenotype of *cdc25-22*, suggesting that Ppa2 and Wee1 may function in parallel.

In higher eukaryotes, PP2A is sharply downregulated during mitosis by greatwall kinase through its target proteins, alpha-endosulphine and Arpp19, which are inhibitors of PP2A [42,43]. Greatwall kinase phosphorylates and activates the inhibitors of the PP2A-B55delta holoenzyme. In *S. pombe*, homologues of greatwall and Arpp19/endosulphine are Ppk18 and Mug134, respectively, judging from the database search, whereas in *Saccharomyces cerevisiae*, RIM15 kinase and IGO1/2 represent the counterparts, respectively [44]. RIM15 is known to phosphorylate IGO1, which is required for initiation of G0 phase. It is of considerable interest whether these budding yeast and fission yeast counterparts of greatwall and alpha-endosulphine (and/or Arpp19) also negatively regulate PP2A homologues during mitosis. In *S. pombe*, the B55delta counterpart subunit is probably Pab1. The *Δpab1* deletion phenotype suggests that Pab1, the regulatory subunit of PP2A, may control the polar actin distribution [45], possibly linked to cell shape control. Our recent results indicate that *Δppe1*, *Δekc1* and *Δpab2* lose their viability in nitrogen-starved G0 cells (K. Sajiki 2011, unpublished data), suggesting that these phosphatases may also be involved in cell size and shape change under nitrogen starvation.

## 7. Sds23 is related to diverse functions by binding to and inhibiting PP2A and PP2A-related phosphatases

**Figure 3.**
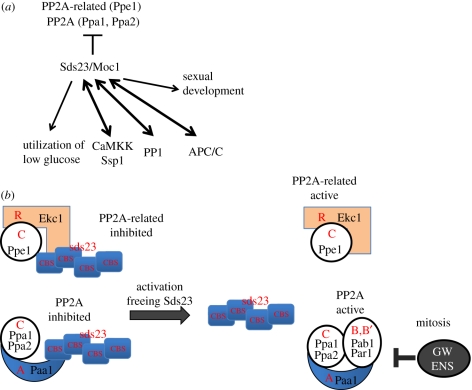
Diverse roles of Sds23. (*a*) High-copy plasmid carrying the *sds23*^+^ gene suppresses the mutations of *ssp1*, protein phosphatase PP1 and APC/cyclosome [21,46,51]. Sds23/Moc1 is required for the utilization of low glucose and sexual development. (*b*) Sds23 stably associates with PP2A-related Ppe1–Ekc1 and PP2A phosphatases (see text). C, R: catalytic and regulatory subunits of PP2A-related phosphatase. C, A, B: catalytic, regulatory A and B subunits of PP2A. The phosphatase free from Sds23 seems to be active [21]. GW and ENS represent greatwall kinase and alpha-endosulphine, respectively (see text).

Hanyu *et al*. [21] reported that Sds23 is a key to linking phosphatases with the utilization of low glucose and related kinase Ssp1. Sds23 was identified as one of the three high-copy suppressors for cs *dis2-11* that is defective in PP1 phosphatase [46]. The remaining two other suppressors are Sds21, the second PP1 catalytic subunit [47,48], and Sds22, the positive regulatory subunit of PP1 to promote metaphase–anaphase progression [49–51]. The mammalian homologue of Sds22 is implicated in cancer [52]. Sds23 is known to be related to diverse functions through its ability as a high-copy suppressor for mutants of PP1, APC/cyclosome subunits, Ssp1 and others (figure 3*a*; [21,46]; Y. Hanyu & M. Yanagida 2011, unpublished data). Sds23 is also involved in inducing sexual development as Moc1 is identical to Sds23 [53]. Conversely, high-copy plasmids carrying the PP1 *dis2*^*+*^, APC/C *cut9*^*+*^ and *ssp1*^+^ gene rescue the deletion of Sds23, so that the suppression is reciprocal (figure 3*a*).

A critical finding to understand these diverse functions of Sds23 is that Sds23 binds to PP2A and PP2A-related phosphatases, and inhibits *in vitro* the PP2A-related phosphatase activity of Ppe1 [21]. Analyses of mass spectroscopy and two-hybrid interactions demonstrate that Sds23 is bound directly to the regulatory subunits Ekc1 (SAP-like) and Paa1 (the subunit A-like, figure 3*b*; [21]). As the B and B′ subunits (Pab1 and Par1, respectively) of PP2A are scarce or missing in the immunoprecipitates, Sds23 might associate with an intermediate assembly form of PP2A holoenzymes. Taken together, high-copy suppression of many mutations by Sds23 appears to be due to the collective negative modulation of two major phosphatases, PP2A and PP2A-related phosphatases, through direct inhibition of the regulatory subunits Paa1 and Ekc1. The inhibitory role of Sds23 is reminiscent of the PP2A inhibitor alpha-endosulphine (IGO1/2, Mus134) that is the target of greatwall kinase. However, Sds23 acts in interphase or throughout the cell cycle. Homologues of Sds23 are found in all fungi and in cellular slime mould, but not in higher eukaryotes so far [21]. It is similar to, but distinct from, the γ subunit of AMPK: both Sds23 and AMPK γ subunit contain the two cystathionine-β-synthase domains.

Sds23 is required to use low glucose: the deletion mutant *Δsds23* fails to proliferate in 0.1 per cent glucose and slowly consumes glucose [21]. In *sds23*-deficient cell extracts, the phosphatase activity greatly increases, and is diminished by the addition of okadaic acid, an inhibitor of PP2A and related phosphatases. The high phosphatase activity of PP2A and PP2A-related may hence be inhibitory to use low glucose. Ssp1 and the phosphatases may be opposing, and Sds23 and Ssp1 synergistically cooperate to use low glucose. The downregulation of PP2A and PP2A-related phosphatases appears to be required for using the low concentration of glucose in the culture medium.

## 8. Reverse cell size phenotypes of *tor1* and *tor2* mutants

**Figure 4.**
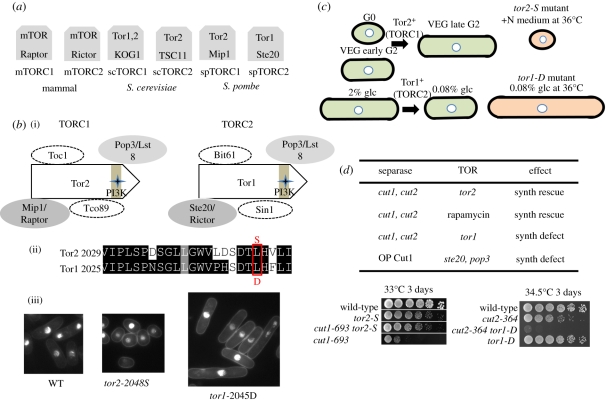
Functional relationship of TORCs with securin and separase. (*a*) Two TORCs exist in mammals, *Saccharomyces cerevisiae* and *Schizosaccharomyces pombe*. Mammalian TOR is the sole catalytic subunit in mammals, while budding and fission yeasts have two distinct catalytic subunits. (*b*)(i) The subunit constituents of TORC1 and 2 in *S. pombe*. The crosses indicate the mutation sites of *tor1-D* and *tor2-S* in the PI3K domain of Tor1 and Tor2. (*b*)(ii) The mutated residues are indicated in red colour in the conserved amino acid sequences in the PI3K domain. (*b*)(iii) DAPI-stained micrographs of wild-type, *tor2-S* and *tor1-D* cultured at 36°C for 6 h. (*c*) Schematic of the role of Tor2 and Tor1 in cell size determination (see text). glc, glucose. (*d*) Synthetic rescue or defect observed in the pair of mutations in securin/*cut2*, separase/*cut1* or overproduction (OP) of Cut1 and mutations of *tor1*, *tor2*, regulatory subunits (*ste20* and *pop3*) or the addition of rapamycin. See text. Two examples of the spot test at the semi-permissive temperature, showing the (left) synthetic rescue and (right) defect, are shown (see text).

The target of rapamycin complex (TORC) 1 and 2 exist in eukaryotes (figure 4*a*; [54]). A variety of cell functions involved in cell growth in response to nutritional cues are controlled by TORCs: TORCs are thought to be the central regulators of growth upon nutritional alterations. Rapamycin is an antiproliferative drug that prolongs the life of model animals, and might be useful in the treatment of certain cancers. Rapamycin bound to FKBP12 (a peptidyl–prolyl *cis*–*trans* isomerase) inhibits TORC1 [55]. The mammalian TOR is the sole catalytic subunit, whereas *S. cerevisiae* and *S. pombe* contain two highly similar catalytic subunits, Tor1 and Tor2 (figure 4*a*).

*Saccharomyces cerevisiae* TORC1 and TORC2 mediate the control of many cellular events [56–61]. The C-terminal domain of catalytic subunits contains a lipid kinase motif, which places them in the phosphatidyl inositol-kinase-related kinase (PIKK) family [62]. Tor1 and Tor2 form two functionally distinct TOR complexes [58]. TORC1, which is responsible for many of the known TOR functions [63], contains either Tor1 or Tor2 (figure 4*a*). TORC2, which is not sensitive to rapamycin, helps regulating actin cytoskeleton polarization [60], and its complex function is less understood.

Similar to *S. cerevisiae*, *S. pombe* has two TOR kinase genes, *tor1*^+^ and *tor2*^+^ [34,64–68]. However, the nomenclature of TOR kinase in *S. pombe* is unfortunate; *S. pombe* Tor2 is similar to *S. cerevisiae* Tor1, whereas *S. pombe* Tor1 is similar to *S. cerevisiae* Tor2. Accordingly, TORC1 and TORC2 in *S. pombe* contain distinct catalytic subunits, Tor2 and Tor1, respectively (figure 4*a*,*b*). Mass spectrometric analysis with immunoprecipitation experiments indicated that *S. pombe* TORC1 contains only Tor2 [20,34].

To couple extracellular nutrient signals with cell growth, *S. pombe* TORC1 and TORC2 are reported to be controlled by the small GTPases, Rheb1 [69] and Ryh1 [70], respectively. Wild-type *S. pombe* is insensitive to rapamycin [71]. However, *S. pombe* becomes sensitive to rapamycin under conditions of starvation [72] or in *tor2* mutants [34].

Each of *S. pombe* TORC1 and TORC2 contains four evolutionarily conserved regulatory subunits (figure 4*b*; [34]). Mip1 and Ste20 are homologues of mammalian Raptor and Rictor, respectively, while Pop3/Wat1 is a homologue of Lst8 that associates with both TORC1 and TORC2. The mutation site of a ts and rapamycin-sensitive *tor2-S* is the substitution from L2048 to S (figure 4*b*(ii); [34]). A new ts mutant *tor1-D* was constructed by introducing the D (aspartate) residue at the conserved 2045L as described above. This *tor1-D* mutant is useful for critical comparison between *tor1* and *tor2* phenotypes as the mutation sites are basically identical (the inset of sequences; [20]). The cellular phenotypes of *tor1-D* and *tor2-S* at 36°C in the complete medium are virtually opposite (figure 4*b*(iii); [20]). The short, ellipsoidal-shaped phenotype was produced by *tor2-S*, whereas the elongated cells were observed for the *tor1-D* mutant, suggesting that their mode of involvement in the cell size control, and possibly also in their relation to the cell cycle, may be opposing.

## 9. Nutrient-dependent cell size control by Tor1 and Tor2 kinases

The opposite phenotypes of *tor1-D* and *tor2-S* in cell size may be explained by the different roles of Tor1 and Tor2 in responding to nutritional signals (figure 4*c*). As the currently held view on TORC1 [73], Tor2 facilitates the intracellular use of nitrogen source and supports growth (the increase of cell volume). In *tor2-S* mutants even in the ample presence of environmental nitrogen source, protein and nucleic acid synthesis become defective, resulting in the inhibition of growth, but one or two rounds of the size-shortening divisions occur, reminiscent of the wild-type cell behaviour in N-starvation. If *tor2-S* cells that arrested in the G0 phase at 26°C are shifted to 36°C in the presence of nitrogen source, then cells do not exit from the G0 phase [34]. These results indicate that the small cell size of *tor2-S* at 36°C is due to the residual rounds of cell division in the absence of growth. As this phenotype at 36°C suggests, Tor2 is surely required for growth, but it is uncertain how it relates to cell cycle control.

While Tor2 is essential, Tor1 is dispensable. The phenotype of *tor1-D* is becomes clearly defective under low glucose (less than 0.1%): *tor1-D* mutant is defective in shortening cell size under low glucose, like *ssp1* mutants (figure 4*c*). The failure to reduce the cell length under glucose limitation leads to semi-*cdc25*-like cell elongation for *tor1-D* [20]. Tor1 and Ssp1 may thus sense low glucose and reduce the cell length through regulating mitosis and cytokinesis. In this regard, Tor1 is like a cell cycle-controlling gene. Indeed, the genetic interaction of the deletion *Δtor1* with *cdc25* mutation was reported [74,75].

The timing of cell cycle events in *tor1-D* after the release from the G0 phase was monitored. DNA replication occurred with the same timing as the wild-type, but the entry into mitosis and subsequent cytokinesis is delayed, resulting in the increase of cell size [20]. The delay is due to the delay in Cdc2 mitotic activation, as Y15 phosphorylation of Cdc2 remains in elongated cells. Interestingly, actin was abundant at the single cell tip in the interphase of *tor1-D* mutants. Bipolar cell elongation seems to be absent in *tor1-D* mutant cells. The intensity of actin at the equator was also high, whereas myosin makes a normal-looking contractile ring. Interphase monopolar growth of *tor1-D* mutants that accompanied abundant actin localization at one single tip might cause the delay in Cdc2 activation. Proper localization of actin may be required for signalling the transition of nutrient state or utilization, which may be the upstream event for regulating the G2–M transition.

## 10. Requirement of separase/Cut1-securin/Cut2 is alleviated in tor2-diminished cells

Surprisingly, the complex of securin/Cut2–separase/, essential for chromosome segregation, strongly interacts with both TORC1 and 2. Alleles of *cut1* were synthetically rescued by *tor2-S* mutation (one example of the spot test is shown in figure 4*d*; [20]). The addition of rapamycin to *cut1* mutants also strongly suppressed the ts phenotype. As the main target of rapamycin is Tor2 in *S. pombe*, these results suggest that the downregulation of Tor2 lessens the necessity for Cut1. Consistently, the synthetic rescue by *tor2-S* mutation and rapamycin was also found for the mutant alleles of *cut2*. As the levels of Cut1 and Cut2 are not restored at all in cells suppressed by *tor2-S* or rapamycin, the necessity of Cut1–Cut2 is greatly alleviated, or the mode of chromosome segregation under the diminished Tor2 situation may be drastically altered, regarding the requirement of Cut1–Cut2. It remains to be determined which of the Cut1 functions that include proteolytic and non-proteolytic activities in *S. pombe* [64,65,76] are actually suppressed.

The additive defects were observed between *cut2* and *tor1* (the spot test result is shown in figure 4*d*). Between *cut1* and *tor1*, the additive effect also exists. These results suggest that the Cut2–Cut1 complex shares the essential function with Tor1. The synthetic defect was also found between the overproduction of Cut1 and the *tor1-D* mutant. The previous study [77] showed that overproduction of Cut1 causes the synthetic defect with *ste20* and *pop3* mutations that are defective in the regulatory subunits of TORCs. These apparently opposite results between Tor1 and Tor2 in the interaction with Cut1–Cut2 mutations again suggest that the roles of Tor1 and Tor2 are opposing (discussed below).

Other unexpected finding is that the ts phenotype of the *cut2* mutant is partly suppressed by *Δfkh1*, the deletion of FKBP12-like Fkh1, in the absence of rapamycin [20]. Fkh1 encodes a peptidylproline *cis*–*trans* isomerase enzyme, which accelerates the folding of proteins. While Fkh1 is necessary for *tor2-S* and *tor1-D* mutants to be sensitive to rapamycin, suppression of the ts phenotype of *cut2* by the deletion *Δfkh1* occurs in the absence of rapamycin. Fkh1 appears to be needed for the ts phenotype of the *cut2* mutant. Since Fkh1 affects the protein conformation, the result might fit with a notion that Cut2 is a chaperone-inhibitor of Cut1 [54]: Fkh1 may cause instability of mutant Cut2 protein.

## 11. Discussion

The aim of this review is to discuss the perspective regarding facts and hypotheses on size control during the cell division cycle under limited nutrients and their implication in the mode of mitosis. First, it should be emphasized that cell size control is the meeting point for cell division cycle and growth control. When considering growth control, it is not surprising that glucose and nitrogen source are determinant factors for the cell size. The cell length of *S. pombe* at the time of division is pre-determined, depending on different concentrations of nitrogen source and glucose in the culture medium. The *wee* or semi-*wee* cellular phenomenon occurs in wild-type cells under limited nutrients. Second, protein phosphorylation and dephosphorylation are closely implicated in the cell size control. Several kinases and phosphatases are found to control cell size under the nutritional limitations. Discussions are mostly restricted to the cases of *S. pombe* rather than budding yeast and mammalian systems. The reason is that critical examination of mammalian cell size control during the somatic cell division cycle has been scarce and that the role of SWE1 (Wee1 homologue) and MIH1 (Cdc25 homologue) in cell size control at the time of *S. cerevisiae* division is unclear.

As is the case of *S. pombe* Cdc25 and Wee1, which are opposing phosphatase and kinase, but actually coordinate the timing of Cdc2 activation for mitotic entry through the change of their activities, TORC1 (Tor2) and TORC2 (Tor1) may be opposing, but actually coordinate growth, mitosis and cell size control in response to nutritional cues. Premature mitosis is observed in the *tor2-S* mutant, whereas cells are elongated in the *tor1-D* mutant, reminiscent of *wee1* and *cdc25* mutants. TORC1 and TORC2 are both kinases, so that they are unlikely to target the same substrate to control the cell size by their opposing functions. It is possible that TORC1 and TORC2 are the upstream regulators for Cdc2 activation, and affect directly or indirectly Wee1 and Cdc25 depending on the levels of nutrients. Note that *S. pombe* has the second Wee1-like kinase, Mik1, which may also be involved in the nutritional control for Cdc2 activation. The hypothesis that TORC1 and TORC2 control Cdc2 activation at the G2–M boundary in opposing manners remains to be tested. Nutrient-sensitive Ssp1 kinase (and possibly also Ssp2 AMPK) and PP2A (Ppa2), PP2A-related (Ppe1) phosphatases are also opposing. The Ssp1–Ppa2/Ppe1 signalling may also be considered as the upstream regulators for Cdc2 activation. They are implicated in calcium signalling in addition to nutrient uptake. However, their relationship to TOR kinases, dependent or independent, remains unclear. The Ssp1–Ppa2/Ppe1 signalling may target the same substrate(s) to control the cell size under different nutrients.

We argue that Sds23 is a key molecule to understand the role of Ppa2 and Ppe1 phosphatases for the nutritional utilizations because Sds23 restrains the phosphatases by stable binding. The loss of Sds23 leads to the hyperactivation of the phosphatases, causing the failure to consume the low concentration of glucose for cell proliferation [21] and resulting in loss of viability of the G0 cells under nitrogen starvation [14]. The effect of *Δsds23* deletion on the glucose consumption is exceptionally strong among thousands of mutant strains examined. Measurement indicates that the consumption rate of glucose in *Δsds23* cells is extremely slow (L. Uehara & A. Mori 2011, unpublished data). The biomarker compound cytidine diphosphate-choline, the level of which greatly increases under glucose fasting in *S. pombe* [33], considerably increases in the extracts of *Δsds23* deletion mutants (T. Pluskal 2011, unpublished data), strongly suggesting that the intracellular nutrient state of *Δsds23* is close to glucose starvation, whereas glucose is abundant in the culture medium. The uptake of low glucose is clearly defective in Δ*sds23*. In higher glucose concentration (2%), *Δsds23* cells can proliferate, suggesting that the glucose transport becomes very inefficient. We consider that this kind of analysis might be useful for understanding the human disease type II diabetes.

The cell length of *Δsds23* is long and rod-shaped [46], resembling the overproduction phenotype of Ppa2 and Ppe1 phosphatases. Sds23 is highly phosphorylated. Judging from the phosphorylated residues determined by mass spectrometry, candidate kinases are PKA, protein kinase C and MAPK. It is important to determine whether phosphorylation controls the binding to phosphatase. We consider that Sds23 acts in parallel with PP1 and APC/cyclosome, and negatively regulates PP2A and PP2A-related phosphatases in interphase or throughout the cell cycle. The role of Sds23 or the downregulation of PP2A and PP2A-related phosphatases becomes essential when the levels of glucose and nitrogen source are low. The transcript level of Sds23 sharply increases by oxidative (H_2_O_2_) stress, and transiently increases by cadmium, heat and sorbitol treatments [78].

The role of PP2A in cell size control and its interactions with Cdc25 and Wee1 was documented many years ago in *S. pombe* [22,23,41], but their negative role for glucose consumption has only recently been realized through the study on Sds23. Furthermore, the discovery of mitotic inhibition of PP2A by phosphorylation of alpha-endosulphine and Arpp19 by greatwall kinase [42,43] strongly suggested the importance of PP2A downregulation during mitotic progression. It is conceivable that the downregulation of PP2A and PP2A-related phosphatases during mitosis enhances the utilization of glucose and the maximal production of energy source.

The role of CaMKK-like Ssp1 kinase is similar to that of Sds23. Ssp1 is essential to properly respond to the starvation of nitrogen source and also glucose [13,14,21], suggesting that Ssp1 may broadly contribute to survival under limited nutrients. However, its molecular function, particularly in the relation to calcium and calmodulin, has been little studied. It is scarcely understood how actin localization, and glucose and nitrogen source utilization are integrated with Ssp1 kinase. The short, conserved stretch sequence present between the kinase- and calmodulin-binding domains is essential for maintaining rod-like cell shape [21]. Mass spectrometric analysis shows that Ssp1 is bound to 14-3-3 homologues, Rad24 and Rad25, suggesting that Ssp1 shuttles between the nucleus and cytoplasm. Ssp1 is highly phosphorylated: seven phosphopeptides have been identified [21]. Overproduction of Ssp1 disperses cortex actin, resulting in the production of round cells. By contrast, under nitrogen starvation, *ssp1* mutants keep the rod cell shape and fail to reduce cell size. The role of Ssp1 based on the interaction with Sty1 and Wis1 is of considerable interest. Our knowledge is still scarce for the relationship between Ssp1 and Sty1 in the regulation of cell size under nitrogen starvation.

To critically compare the phenotypes between *tor1* and *tor2* mutants, the ts mutant *tor1-D* that has the substitution at the same PI3K site as that of the previously isolated *tor2-S* mutant is useful [20]. Their phenotypic comparison indicates that Tor1 and Tor2 are opposite in many aspects including the cell size and also in the mode of interactions with securin and separase mutations. Rapamycin is highly inhibitory to *tor2-S*, but only slightly to *tor1-D*. As mutant TORC1 and TORC2 kinases are purified and their TOR kinase activities are found to be greatly diminished [20], different phenotypes may be due to the substrate specificities of TORCs kinases.

We interpreted that the opposite cell size phenotypes of *tor1-D* and *tor2-S* are due to their distinct responses to nutritional conditions. One important role of Tor1 is to reduce the cell size for division upon decrease of glucose concentration. Effects of other nutrients on the cell size control by Tor1 remain investigated. By contrast, the role of Tor2 is to support growth by increasing the cell size until cells reach the critical size for division. Hence, Tor1 and Tor2 may be considered to coordinate growth with the determination of the timing of cell division depending on nutritional conditions, although their apparent phenotypes and deduced functions look to be opposing. To substantiate such a hypothesis, more mechanistic studies are necessary in future, particularly, in relation to Cdc2 activation as discussed above.

The final issue is how to interpret the synthetic rescue of *cut1* and *cut2* mutations by *tor2-S* or rapamycin. The suppression by rapamycin requires Fkh1, a FKBP homologue. This unexpected rescue is strong so that the functional loss of Cut1–Cut2 complex is clearly alleviated if Tor2 (TORC1) is diminished. Chromosome segregation looks quite normal in the suppressed mutant cells, but the low level of separase and securin does not increase at all, as if only a small amount of Cut1–Cut2 is needed in *tor2* or rapamycin-treated cells [20]. The complex of Cut1–Cut2 is absolutely needed for proper chromosome segregation in wild-type cells cultured in the regular medium, but the necessity is known to be greatly lessened in the *cut1* and *cut2* mutant cells in medium containing high concentrations of salt or sorbitol [64]. However, effect of salt differs from that of rapamycin, as the high salt increases the level of Cut1–Cut2 in a Sty1-dependent manner. It is well known in mammalian cells that immunological response (transplant rejection) is suppressed after rapamycin treatment. Additionally, the progression of certain cancers is prevented, and the lifespan is extended in mice. Although the mechanism is scarcely understood, diminished Tor2 (TORC1) greatly lessens the necessity of both Cut1 and Cut2, suggesting that the mode of chromosome segregation might be dramatically altered by the nutritional change via Tor2 (TORC1).

Our results suggest that the segregation role of Cut1–Cut2 is opposite to TORC1 (Tor2), but in parallel with TORC2 (Tor1). There is a popular concept that growth opposes cell division. Conversely, in *S. pombe*, the period of mitosis restrains growth (cell elongation). TORC1 may restrain the Cut2–Cut1 function in order to prevent premature mitosis, while TORC2 is in parallel with Cut2–Cut1 as its role is to determine the critical timing of division under different nutritional conditions. The close interaction of the TORC complexes with Cut2–Cut1 suggests that nutritional control may be mediated at the metaphase–anaphase transition. This is a surprising possibility, and calls for further study. The nutritional control might be exerted on the metaphase–anaphase progression through the functions of Cut1–Cut2, which might require the balanced regulation of TORC1 function. Cut2 is phosphorylated ([64]; Y. Hanyu & M. Yanagida 2011, unpublished data). Further study is definitively needed for understanding these interactions of Cut1–Cut2 with TORC1 and TORC2.

In summary, we identified Sty1, Wis1, Ssp1, Tor1 and Tor2 kinases, and Ppa2 and Ppe1 protein phosphatases to be important for regulating the cell division cycle control under different nutritional conditions. Their contributions become strikingly apparent in mutant cells under nutritional limitations. These kinases and phosphatases seem to affect Cdc2 kinase activation, though the mechanisms are little understood. Our results revealed the surprising dependency of mitotic metaphase to anaphase transition on the nutrient-sensing TORCs. The block of chromosome segregation by diminished Cut1–Cut2 by mutations is restored by diminished TORC1 by mutation or rapamycin. The mode of chromosome segregation may have to be controlled by TORCs in order to respond to nutritional changes.
